# Nelumbo nucifera Leaf Extract Induces Cytotoxicity in Osteosarcoma Saos-2 Cells

**DOI:** 10.7759/cureus.47609

**Published:** 2023-10-24

**Authors:** Gautam Britina, Devaraj Ezhilarasan, Karthik Shree Harini

**Affiliations:** 1 Dentistry, Saveetha Dental College and Hospitals, Saveetha Institute of Medical and Technical Sciences, Saveetha University, Chennai, IND; 2 Pharmacology, Saveetha Dental College and Hospitals, Saveetha Institute of Medical and Technical Sciences, Saveetha University, Chennai, IND

**Keywords:** saos-2 cells, osteosarcoma, nelumbo nucifera, herbal drug, cytotoxicity, cancer

## Abstract

Background

Osteosarcoma is the eighth most common cancer and its prevalence in children makes it a global concern. Existing medications and treatments like high-dose methotrexate possess harmful side effects. Therefore, novel herbal drugs like *Nelumbo nucifera* are of utmost importance.

Aim

To analyze a novel anticancer herbal drug, *Nelumbo nucifera *leaf extract for its cytotoxic potential against osteosarcoma.

Materials and method

*Nelumbo nucifera* leaf extract was prepared. Saos-2 Cells (human osteosarcoma cell line) were treated with *Nelumbo nucifera* leaf extract (25, 50, 75, 100, 125, and 150 µg/ml) for 24 hours which were then subjected to MTT assay, morphological analysis and DAPI staining.

Results

The results suggested that *Nelumbo nucifera* leaf extract had a concentration-dependent cytotoxic effect on Saos-2 cell line. The extract significantly reduced the number of viable cells, inhibited proliferation and induced morphological changes in Saos-2 cells.

Conclusion

*Nelumbo nucifera* has the potential to induce cytotoxicity against osteosarcoma cell lines and hence, this study provides a novel therapeutic regimen for the treatment of osteosarcoma.

## Introduction

Osteosarcoma, which is also called osteogenic sarcoma, is the most common type of cancer originating in the bones. It is the eighth most common form of childhood cancer accounting for 2.4% of all pediatric cancers and 20% of all primary bone cancers. Long-term survival of osteosarcoma patients can be expected in less than 20% of all other patients who present with or develop overt metastatic disease [1,2]. Osteosarcoma that occurs without any underlying bone pathology or cancer refers to primary osteosarcoma, whereas secondary osteosarcoma is characterized by any underlying bone pathologies. The diagnosis of osteosarcoma involves imaging of the tumor site by radiographs like Magnetic Resonance Imaging, Computed Tomography, nuclear imaging like positron emission imaging and radionuclide bone scans [3,4].

Combined use of high-dose methotrexate, cisplatin and doxorubicin is widely used as the treatment for osteosarcoma [5]. Further, the malignancies associated with osteosarcoma are treated with preoperative and postoperative chemotherapy [6]. Drugs like vincristine, doxorubicin and cyclophosphamide [7] are employed with alternatives of ifosfamide and etoposide [8]. However, the side effects of existing treatment involve risk of infection, nausea, vomiting, hair loss, loss of appetite, kidney damage, myelosuppression, peripheral neuropathy and hypomagnesemia [8] which affects the physical and mental health of osteosarcoma patients under treatment. Moreover, the increased doses of alkylating agents may possibly increase the risk of second malignancies (leukemia) [9]. 

*Nelumbo nucifera* is also called Indian lotus or sacred lotus. It is widely cultivated and extensively used in eastern Asia. Lotus is used as a traditional medicine to treat cough, fever, insomnia, and diarrhea and to balance the body heat [10]. It has several biological properties like antioxidant, antidiarrheal, antiviral, anti-obesity, and hepatoprotective activities. *Nelumbo nucifera, *India’s national flower, can be used for its therapeutic properties because of its cost and availability [11]. The principal bioactive components present in *Nelumo nucifera* are liensinine and nuciferine, which have been proven to show many medicinal properties with the ability to treat diseases including cancer [12]. There are various reports exhibiting the anticancer property of *Nelumbo nucifera*. *Nelumbo nucifera* has been demonstrated to inhibit the proliferation and metastasis of breast, colon and non-small cell lung cancer cell lines. Particularly, a recent *in vitro* study on HCT-116 (a human colorectal cancer cell line) proved that *Nelumbo nucifera* stamen extract promoted apoptosis of the cancer cells [13]. Therefore, the objectives of this study are to analyze the antiproliferative activity of *Nelumbo nucifera* leaf extract against Saos-2 cells and to examine *Nelumbo nucifera* leaf extract-induced cellular changes in Saos-2 cells for the treatment of osteosarcoma. 

## Materials and methods

Reagents and chemicals

Dulbecco’s minimum-low glucose medium (DMEM), penicillin, streptomycin, trypsin-ethylenediaminetetraacetic acid (EDTA), fetal bovine serum (FBS) and 3-(4,5-dimethylthiazol-2-yl)-2,5-diphenyltetrazolium bromide (MTT) were purchased from Gibco (Billings, MT, USA). All other necessary chemicals were of analytical grade and purchased locally.

Plant extract preparation

Leaf powder of *Nelumbo nucifera* was obtained from IMPCOPS (Chennai, India). To prepare the extract, 50 g of *Nelumbo nucifera* leaf powder was soaked in 95% ethanol for three days at room temperature. The solution was subjected to two steps of filtration using a crude filter paper and a Whatman paper. The filtrate was then placed in a rotary evaporator. About 3g of material was obtained after evaporation. Concentration of the extract was carried out in a vacuum evaporator and stored at 4˚C for further use.

Cell culture

Saos-2 (Sarcoma osteogenic) cell line was procured from the National Centre for Cell Science, Pune, India. The cells were cultured in DMEM with 10% FBS, penicillin (100 units/mL), and streptomycin (100 μg/mL) at 5% CO_2_ and 37^o^C. The cells were treated with 25, 50, 75, 100, 125, and 150 µg/ml of *Nelumbo nucifera* extract for 24 hours.

MTT assay

Following treatment with *Nelumbo nucifera *leaf extract of different concentrations, the cells were treated with MTT reagent, and the cells were incubated in a humidified incubator (5% CO_2_) at 37°C to allow the MTT to be metabolized. After four hours of incubation, the formazan crystals formed were dissolved with dimethyl sulfoxide. Absorbance of the samples was measured at a wavelength of 540 nm using a microplate reader.

DAPI staining

According to Dmitrieva and Burg, 2008 [14], the cells treated with *Nelumbo nucifera* leaf extract at different concentrations for 24 hours were stained with 4,6-diamidino-2-phenylindole (DAPI). The fluorescence was measured at 405 nm, the cells were observed under a fluorescent microscope and images were captured. 

Statistical analysis

Data were expressed as mean ± SD and analyzed by Dunnett’s test following one-way ANOVA. A *p* value lower than 0.05 was considered to be significant.

## Results

**Figure 1:**
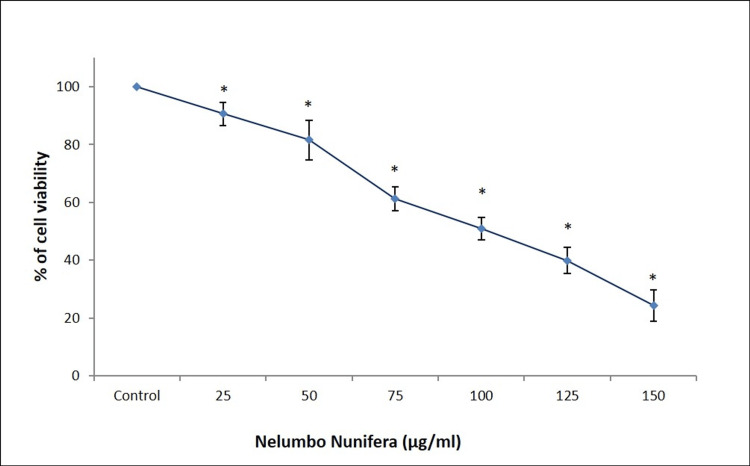
Effect of Nelumbo nucifera against Saos-2 cell viability by MTT assay Dose-dependent decrease in Saos-2 cell proliferation after 24 hours of treatment with *Nelumbo nucifera* leaf extract. Data are shown as means ± SD (n = 3) compared with the control group, ^*^p < 0.001. Saos-2: human osteosarcoma cell line, MTT: 3-(4,5-dimethylthiazol-2-yl)-2,5-diphenyltetrazolium bromide

The cytotoxic effect of *Nelumbo nucifera *leaf extract on the fibroblast cells was determined by MTT assay. Cells were treated with 10, 20, 30, 50, 100, 125, and 150 μg/ml of *Nelumbo nucifera* leaf extract for 24 hours. The extract was observed to reduce the viability of Saos-2 cells with increasing concentrations. The proliferation of Saos-2 cells was decreased in a dose-dependent manner with maximum cytotoxicity observed at 150 μg/ml of extract (Figure 1).

**Figure 2:**
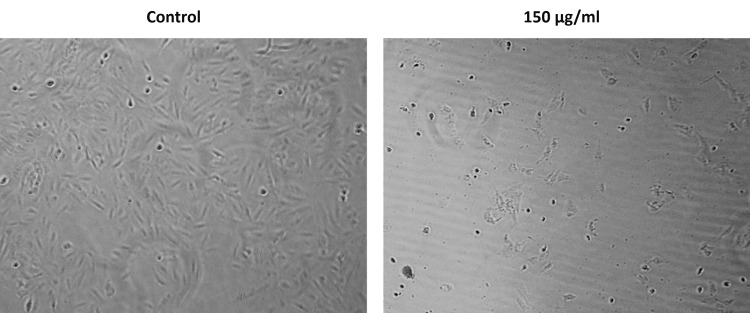
Effect of Nelumbo nucifera on morphological changes of Saos-2 cells Altered morphology of Saos-2 cells and decreased number of viable cells upon 24 hours of treatment of *Nelumbo nucifera* leaf extract. Saos-2 cells: human osteosarcoma cell line

The effect of *Nelumbo nucifera* leaf extract on the cancer cell morphology and viability was analyzed by microscopic analysis. The normal epithelial morphology of Saos-2 cells treated with *Nelumbo nucifera* leaf extract was deformed as evidenced by microscopic observation. Further, a decrease in the number of Saos-2 cells after 24 hours of treatment of *Nelumbo nucifera* leaf extract was observed (Figure 2).

**Figure 3:**
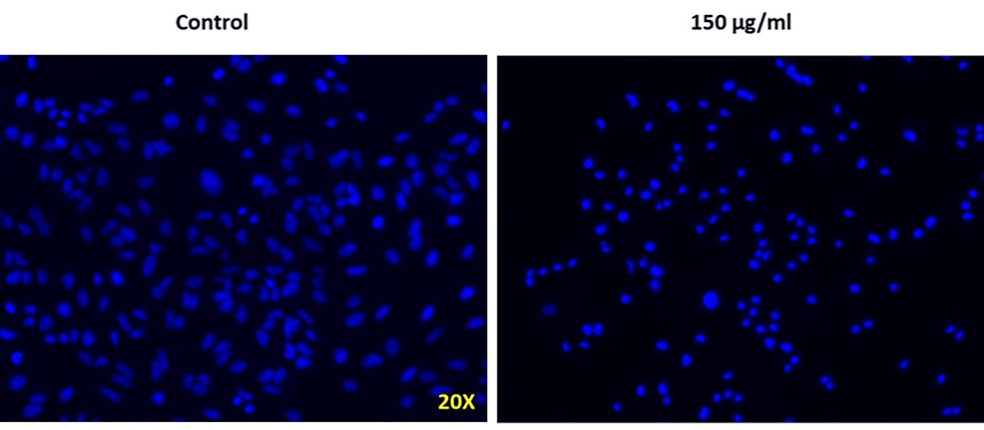
Effect of Nelumbo nucifera on nuclear changes of Saos-2 cells *Nelumbo nucifera* treatment on Saos-2 cells resulted in fragmented nuclei which led to cell death. Saos-2: human osteosarcoma cell line

DAPI staining was performed to analyze the *Nelumbo nucifera* leaf extract-induced morphological changes in the nucleus of Saos-2 cells. Chromatin condensation and nuclear fragmentation leading to cell death confirmed the cytotoxic effect of *Nelumbo nucifera* leaf extract on Saos-2 cell line (Figure 3).

## Discussion

The prevalence of osteosarcoma is high, especially in children between the ages of 10 and 19, and in adults above the age of 65, which makes it a global threat. Although therapeutic medications are prescribed and implemented for the treatment of osteosarcoma, the limitations of these medications and novel therapeutic approaches are widely studied. Accordingly, various herbs and herbal compounds were examined for their anticancer properties against osteosarcoma. Herbs such as *Rabdosia rubescens, *Baikal skullcap(*Scutellaria baicalensis Georgi*)*, Olea europaea L., and Evodia rutaecarpa* and their components have been investigated for anti-cancer effects [15]. Furthermore, all the parts of *Nelumbo nucifera* like the leaves, rhizome, flowers and seeds were considered to be of medicinal importance traditionally [16]. On that account, *Nelumbo nucifera* leaf was analyzed in this study against a human osteosarcoma cell line. 

According to Zhao et al. [13], *Nelumbo nucifera* stamen extract may be a potent anticancer agent against colon cancer. The study revealed that *Nelumbo nucifera* stamen extract prevented cancer cell growth in a dose-dependent fashion. The extract induced apoptosis of HCT-116 cells by upregulating the apoptosis-associated mRNA expression of Fas, Fas ligand, death receptor-4 and 5, caspases-3,8 and 9. Consistent with the above reports, our study also showed that *Nelumbo nucifera* leaf extract exhibits concentration-dependent cytotoxicity against the Saos-2 osteosarcoma cell line, evidenced by the decreased viability of cells in the MTT assay. Further, the reduced number of viable Saos-2 cells under microscopic observation revealed the anti-proliferative effect of *Nelumbo nucifera* leaf extract. 

Plant bioactive components such as curcumin, butein, berberine, quercetin, silibinin, galangin, resveratrol, etc. have been shown to suppress osteosarcoma [17]. There are two strategies bioactive substances employ to target cancer cells [18]. They function as cytotoxic substances that target macromolecules like DNA, enzymes, and microtubules within the cancer cells. Another anticancer strategy of bioactive compounds involves targeting the oncogenic signal transduction pathways that regulate the cancer progression. Correspondingly, alkaloids, flavonoids, phenols, tannins, steroids and glycosides are the major constituents responsible for the biological properties of *Nelumbo nucifera* [19]. Liensinine and nuciferine, present in *Nelumbo nucifera*,were reported to* *inhibit the receptor activator of nuclear factor kappa-B ligand-induced osteoclast differentiation in mouse bone marrow macrophage cells and mature osteoclast-mediated bone resorption which inhibits the growth of breast cancer cells and breast cancer-associated bone loss [20]. Among the various mechanisms of anticancer agents, apoptosis induction is known to play a major role. Several cellular, nuclear and molecular changes occur during apoptosis. Chromosomal fragmentation and condensation have been reported as crucial events of apoptosis. *Nelumbo nucifera* leaf extract induced DNA fragmentation in the rat aortic smooth muscle cell line A7r5 causing apoptotic cell death [21]. Similarly, DAPI staining in our present study demonstrated that *Nelumbo nucifera *leaf extract resulted in apoptosis of Saos-2 cells characterized by fragmentation and condensation of chromosomes. 

Collectively, anticancer agents including herbs and herbal constituents predominantly target cancer cell proliferation, migration and angiogenesis [22]. The results of our study suggested that *Nelumbo nucifera* leaf extract potentially inhibits the cell survival and proliferation of Saos-2 cells by promoting apoptosis. These anticancer properties of *Nelumbo nucifera can be* attributed to the presence of bioactive components such as liensinine and nuciferine. Since herb- and plant-formulated drugs show better antitumor effects and lesser to no adverse effects when compared with chemotherapeutic drugs, our study provides a novel therapeutic candidate for the treatment of osteosarcoma [23]. Additionally, *Nelumbo nucifera* leaf extract could be a potential anticancer agent, which requires extensive studies. 

Limitations

This study demonstrates that *Nelumbo nucifera* leaf extract has the ability to inhibit proliferation, reduce viability and promote cell death of Saos-2 cells and further induce nuclear changes. However, a detailed phytochemical analysis of the components of the extract and the exact mechanism in which the extract and its components exert its anticancer effect is yet to be determined. Moreover, analyzing the molecular level changes induced by the extract will enhance the understanding and application of *Nelumbo nucifera* leaf extract in the osteosarcoma treatment. 

## Conclusions

The study concluded that *Nelumbo nucifera* leaf extract had a concentration-dependent cytotoxic effect on Saos2 cell lines. *Nelumbo nucifera* extract possesses cytotoxic and anti-proliferative properties against Saos-2 cells. Therefore, it might be used against osteosarcoma after necessary *in vivo* and clinical studies.

## References

[1] (2020). Current Advances in the Science of Osteosarcoma: Research Perspectives: Tumor Biology, Organ Microenvironment, Potential New Therapeutic Targets, and Canine Models. Current advances in the science of osteosarcoma: Research perspectives: Tumor biology, organ microenvironment, potential new therapeutic targets, and canine models.

[2] Aljubran AH, Griffin A, Pintilie M, Blackstein M (2009). Osteosarcoma in adolescents and adults: survival analysis with and without lung metastases. Ann Oncol.

[3] Bukhari MH, Qamar S, Batool F (2019). Differential diagnosis of osteogenic tumors in the context of osteosarcoma. Osteosarcoma - Diagnosis, Mechanisms, and Translational Developments.

[4] Shree Harini K, Ezhilarasan D, Lakshmi T (2022). Novel fibroblast growth factor receptor inhibitors: potential therapeutic approach in oral cancer treatment. Oral Oncol.

[5] (2019). Osteosarcoma - Diagnosis, Mechanisms, and Translational Developments.

[6] Asha K, Sachin A, Muzammil S (2012). Chemotherapy in osteosarcoma. Osteosarcoma.

[7] Gheena S, Ezhilarasan D (2023). Personalized mRNA cancer vaccines with immune checkpoint inhibitors: a promising therapeutic approach in oral cancer patients. Oral Oncol.

[8] Rykov MY, Sengapova ER (2019). Treatment of children with osteosarcoma. Osteosarcoma - Diagnosis, Mechanisms, and Translational Developments.

[9] Rheingold SR, Neugut AI, Meadows AT (2003). Therapy-related secondary cancers. Holland-Frei Cancer Medicine. 6th edition.

[10] Wu Y, Wu S, Shi Y (2022). Integrated metabolite profiling and transcriptome analysis reveal candidate genes involved in the formation of yellow Nelumbo nucifera. Genomics.

[11] Zaidi A, Srivastava AK (2019). Nutritional and therapeutic importance of Nelumbo nucifera (Sacred lotus). Era’s J Med Res.

[12] Yasir M, Park J, Han ET (2023). Exploration of flavonoids as lead compounds against Ewing sarcoma through molecular docking, pharmacogenomics analysis, and molecular dynamics simulations. Molecules.

[13] Zhao X, Feng X, Wang C, Peng D, Zhu K, Song JL (2017). Anticancer activity of Nelumbo nucifera stamen extract in human colon cancer HCT-116 cells in vitro. Oncol Lett.

[14] Dmitrieva NI, Burg MB (2008). Analysis of DNA breaks, DNA damage response, and apoptosis produced by high NaCl. Am J Physiol Renal Physiol.

[15] Kazantseva L, Becerra J, Santos-Ruiz L (2022). Traditional medicinal plants as a source of inspiration for osteosarcoma therapy. Molecules.

[16] Chaudhari D, Kiran S, Choudhary A (2023). Prokaryotic communities adapted to microhabitats on the Indian lotus (Nelumbo nucifera) growing in the high-altitude urban Dal Lake. Int Microbiol.

[17] Tobeiha M, Rajabi A, Raisi A (2021). Potential of natural products in osteosarcoma treatment: focus on molecular mechanisms. Biomed Pharmacother.

[18] Imran M, Ullah A, Saeed F, Nadeem M, Arshad MU, Suleria HA (2018). Cucurmin, anticancer, & antitumor perspectives: a comprehensive review. Crit Rev Food Sci Nutr.

[19] He D, Rao X, Deng J, Damaris RN, Yang P (2023). Integration of metabolomics and transcriptomics analyses investigates the accumulation of secondary metabolites in maturing seed plumule of sacred lotus (Nelumbo nucifera). Food Res Int.

[20] Kang EJ, Lee SK, Park KK, Son SH, Kim KR, Chung WY (2017). Liensinine and nuciferine, bioactive components of Nelumbo nucifera, inhibit the growth of breast cancer cells and breast cancer-associated bone loss. Evid Based Complement Alternat Med.

[21] Ho HH, Hsu LS, Chan KC, Chen HM, Wu CH, Wang CJ (2010). Extract from the leaf of nucifera reduced the development of atherosclerosis via inhibition of vascular smooth muscle cell proliferation and migration. Food Chem Toxicol.

[22] Su QH, Xu XQ, Wang JF, Luan JW, Ren X, Huang HY, Bian SS (2019). Anticancer effects of constituents of herbs targeting osteosarcoma. Chin J Integr Med.

[23] Zhang P, Zhang J, Quan H, Chen P, Wang J, Liang Y (2022). Effects of butein on human osteosarcoma cell proliferation, apoptosis, and autophagy through oxidative stress. Hum Exp Toxicol.

